# The Application of Rotational Atherectomy in PCI of Coronary Chronic Total Occlusions

**DOI:** 10.7603/s40602-016-0001-8

**Published:** 2016-01-19

**Authors:** Bin Zhang, Feng Wang, Jack Wei Chieh Tan, Hongtao Liao, Weilu Chai, Huimin Yu, Hong Yan, Lijun Jin

**Affiliations:** 1 section of Cardiology Department, Guangdong Cardiovascular Institute, Guangdong General Hospital, Guangdong Academy of Medical Science, 510080 Guangzhou, China; 2 Department of Cardiology, National Heart Centre Singapore, 5 Hospital Drive, 169609 Singapore, Singapore

**Keywords:** Chronic total occlusion (CTO), Rotational atherectomy, Percutaneous coronary intervention

## Abstract

**Objective:**

The aim of the study is to investigate the effi cacy, complication rate and predictors of rotational atherectomy (RA) usage after successful guidewire crossing during percutaneous coronary intervention (PCI) of coronary chronic total occlusion (CTO).

**Methods:**

A single center experience of 525 consecutive patients from October 2010 to June 2014. A total of 587 CTO lesions were treated. After successful guidewire crossing, lesions that could not be crossed with the smallest 1.25mm balloon underwent RA with 1.25mm and or 1.5mm burrs after exchanging for the Rotawire through a microcatheter. Post RA, the CTO lesions are then pre-dilated successfully before stenting with drug eluting stents (DES). Patients were then clinically followed up for inpatient MACE and restenosis.

**Results:**

587 CTO lesions in 525 patients were treated. The mean age of patients was 61.6 ± 10.2 years-old. 30% had diabetes mellitus. Mean LVEF was 56 ± 12%. The overall successful CTO PCI rate was 87%. 22% required the retrograde CTO approach. 26 CTO lesions in 26 patients (4.43%) underwent RA. RA and stent deployment were successfully performed in 25 patients. One lesion was unsuccessful because the Rotawire could not cross the lesion. Reference lesion diameter was 2.87±0.55mm,18 cases used the 1.25mm burr and 7 cases used the 1.5mm burr. Reference burr / vessel diameter ratio was 0.46± 0.20mm. No patients required adjunct 2b3a inhibitor usage. The procedural success rate was 96.2% and no peri-procedural MACE was observed.

**Conclusion:**

RA was a safe and effective adjunct therapy for calcifi ed CTO lesions that failed balloon dilatation.

## Introduction

Chronic total occlusions (CTOs) are seen in 13-18% of all lesions during coronary angiography and getting to be the most frequent challenge for interventional cardiologists^1^,^2^. Great strides has been made in understanding the pathophysiology of CTO and as well as the development of new CTO PCI techniques, notably antegrade and retrograde PCI methods^3^. However, it remains as the most challenging problem for interventionists with a procedural success rate hovering around 70%. The reason for failure include inability to cross CTO lesions with guide wires in about 80% and failure to cross with balloons catheters in 15% to 20% of cases. Currently, there are not many reports for how to deal with balloon-uncrossable CTO lesions. We studied 26 balloon-uncrossable CTO lesions among a total of consecutively treated 587 CTO lesions at our center from October 2010 to June 2014, which were treated with coronary RA.

## Methods

### Population:

Between October 2010 and June 2014, a total of 525 consecutive patients with 587 CTO lesions at our center were treated with PCI. There were 26 CTO lesions which balloon catheters failed to cross after successful guidewire passing and subs RA was used. Successful balloon dilatations following RA was performed before drug-eluting stents are deployed. Demographic and procedural data for patients with uncrossable CTOs are listed in *Table* 1.

### Definitions:

CTO was defi ned as a complete coronary obstruction (TIMI flow grade 0) with an estimated duration of more than 3 months. Technical success was defi ned as the ability to cross and open the occluded segment with no more than 30% residual stenosis in all views; procedural success was defi ned as technical success with no in hospital MACE, cardiac death, tamponade, coronary artery perforation, and emergency coronary artery bypass grafting. PCI related MI was defi ned by elevation of cTn value (> 5x 99th percentile URL) in patients with normal baseline values (≤99th percentile URL) or a delta change of cTn values > 20% from abnormal baseline values. Hemodynamic instability was defi ned as the occurrence of sustained ventricular arrhythmias or prolonged hypotension (Systolic BP < 90/60 mm Hg).

### Procedure:

Pre-mediation for all patients include a single loading dose of Clopidogrel 300mg given the day before procedure and daily aspirin 100mg. During the procedure, heparin 100 IU/kg was administered. Coronary arteriography and PCI were performed by the right radial artery or femoral artery access. The 26 CTO lesions were crossed by Pilot^®^ 150, Pilot 200 (Abbott Vascular Inc, USA) or Conquest Pro^®^ (Asahi Intec, Japan). When the smallest balloons failed to pass through CTOs, the wires would be taken out via a Finecross^®^ microcatheter (Terumo Company, Japan) or Corsair^®^ (Asahi Intec, Japan) microcatheter. The Rota wire^®^ (Boston Scientifi c Corp) was then exchanged. If the microcatheter failed to cross, the Rota wire^®^ was then manipulated primarily across the CTO. RA (Boston Scientifi c Corp) was performed over the Rota wire^®^, 1.25mm and 1.5mm burrs were used, the rotational speed was between 150000 to 180000 r/min. After successful RA, balloon predilatations were performed before stent implantation.

### Results:

From October 2010 to June 2014, there were a total 587 CTO lesions in 525 patients treated with PCI at the 2nd section of Cardiology Department of Guangdong General Hospital. The overall successful PCI rate was 87%. The majority employed the antegrade approach. 129 CTO cases required the retrograde approach. The success rate was 76.0% for cases that required the retrograde approach. There were 26 patients with balloon-uncrossable CTO lesions which were treated with rotational atherectomy (*Table* 1). Among the 26 CTO lesions, 12 (46.15%) were in the right coronary artery, 10 (38.46%) in left anterior descending artery and 4 (15.38%) in the left circumfl ex artery.

In the 26 CTO-RA procedures, 24 (92.30%) cases employed the transradial approach, while 2 (7.70%) cases used the transfemoral approach. 22 (84.62%) of the CTO lesions were crossed by Pilot 150 wire, 2 CTOs (7.69%) by Pilot 200 wire, and 2 CTOs (7.69%) were by Conquest PRO wire. Parallel wire techniques were used if necessary. The average time of wire crossing through the CTO lesions was (21.38 ± 11.84) min. After failing to cross with the smallest available balloon catheter, such as Ryujin^®^ 1.25×15 mm (Terumo Company) and Sprinter^®^ 1.25×10 mm (Medtronic, Inc.) (*table* 2) in spite of supportive deep seated guiding catheters, double wire support and balloon anchoring techniques, RA was applied. 25 CTOs underwent successful RA. 1 case was unsuccessful as the Rota wire^®^ (Boston Scientifi c Corp) was unable to cross CTO lesion.

The successful rate of rotational atherectomy was 96.15%, and the average procedure time was 89.77 ± 35.83 min. 1 (3.85%) patient developed TIMI II flow after rotational atherectomy and stent implantation. It was treated successfully with intracoronary nitroglycerin 200μg. 1 patient (3.85%) developed transient hypotension requiring intracoronary injection of Aramine (Metaraminol). All the patients had clinical symptomatic improvements after the successful procedure. The average length of stay was (1.85 ± 0.98) days.

**Table 1.** Patients Characteistics

Male	20(76.92%)
Female	6(23.08%)
Age(year)	64±8.1
Prior myocardial infarction	4(15.38%)
Hypertension	14(53.85%)
Diabetes mellitus	8(30.77%)
Dyslipidemia	6(23.08%)
Renal insufficiency	6(23.08%)
Smoking history	6(23.08%)
J-CTO score(total point)^*^	2.92±0.688
Re-try lesion(yes)	4(15.38%)
Entry shape(blunt)	12(46.15%)
Calcification(Presence)	26(100%)
Bending>45°	11(42.31%)
Occlusion length≥20mm	23(88.46%)
NYHA	
Class I	20(76.92%)
Class II	4(15.38%)
Class III	2(7.70%)
Clinical presentation	
Stable angina	12(46.15%)
Unstable angina	14(53.85%)

*category of difficulty(total J-CTO score point): 0-easy, 1-intermediate, 2-difficult, ≥3-very difficult

**Table 2.** Guide catheter and number of balloon catheters used rotational atherectomy

Guiding catheter size and type	
6F	18(69.23%)
7F	8(30.77%)
RCA	JR 10 (83.33%), AL 2 (16.67%)
LCA	XB 12 (85.71%), AL 2 (14.29%)*
Number of balloons used before RA	
1	10(38.46%)
2	14(53.85%)
3	2(7.69%)

RCA: right coronary artery, LCA: left coronary artery

## Discussion

The pathophysiology of CTOs begins with repeated atherosclerotic plaque rupture and thrombosis of the vascular lumen. Over time negative remodeling begins with formation of lesions in nascent capillaries which gradually substitutes the cholesterol rich plaque and protein polysaccharide with collagen fiber and calcium. The end result is a calcified occlusive lesion packed with dense connective tissue^4^. In the majority of patients, the exact vascular occlusion duration is unknown^5^. However, the degree of calcification was positively correlated with occlusion time. 54% of CTO lesions start to show calcification within 3 months and invariably all patients with CTO develop calcification after 5 years in differing degrees^4^.

CTO intervention is increasing becoming standard practice with advances in interventional techniques and equipment. The success rate is high in large volume centers that perform more than 2000 interventions yearly. The bugbear is often the inability to pass any device through long calcified CTO lesions after successful guidewire crossing. In this study, the failure rate to cross these calcified lesions post successful guidewire passage is 4.43% of all CTO lesions attempted. This is reflective of current advances and availability of smaller low profile balloons catheters and various techniques to increase support like the use of strong guide catheter support, double wires support, anchoring balloon technique, deep throating guide engagement, and guideliner catheters etc.

Earlier studies in the pre-stent and bare metal stent era showed that routine debulking with RA did not reduce mortality or target vessel revascularization rate^1^,^6^,^7^. In the current era, RA is done as an adjunct therapy for plaque modification before stenting and not to achieve maximal debulking. The prevailing axiom is that one should go straight to RA whenever heavy calcification is seen on angiography^8^. Some studies has showed that for severely calcific and fibrotic lesions, primary RA can improve success rate by reducing the incidence of intimal tear, stent migration and incidence of stent thrombosis due to appropriate stent apposition and expansion^9^. Investigators from Japan in the DOCTORS study shows that routine pre-stent plaque debulking in CTOs with either RA or directional athrectomy showed favorable mid-term outcome with lower target revascularization rate in the debulking group compared to standard therapy^10^.

The key failure mode to successful RA is passage of the Rota wire. The first step should be an attempt to exchange the guidewire with a microcatheter such as Finecross or Corsair. The micro catheter is placed distal to the CTO lesion, the original wire is removed and replaced with the Rota wire. To increase the success rate of primary Rota wire passing through in heavily calcified lesions that even micro-catheters could not cross, one should try to find the micro-channel created by the 0.014 inch wire. An extra-support Rota wire is preferred for routine RA but a floppy Rota wire is better for primary rotawiring due to the longer tip length. Multiple views on angiography should be done to ensure that the rotational wire is located within the true lumen distal to the lesion before RA. The burr head should proceed slowly during RA. The RA speed is keep between 150000 to 200000 rpm. It is important to avoid deceleration of rotating speed > 5000 rpm with each RA run time kept at less than 20 seconds.

We favor starting with the smallest burr size possible. The main purpose is to perform plaque modifi cation by reducing the calcifi ed tissue and to improve the compliance of vascular wall in order to assist full balloon expansion and complete stent deployment^11^. In the study, 23 cases used the 1.25 mm burr head, while 2 cases used 1.5mm burr head. Only 1 patient with left anterior descending coronary artery proximal occlusion required sequential 1.25mm followed by 1.5mm burr due to insuffi cient debulking. Post RA, we performed predilatation with balloon catheters in a 1:1 reference vessel sizing before stent implantation. Overall, our study is consistent with the low reported rates and safety of RA for CTO intervention^12^,^13^.

## Conclusion

RA is a safe and effective adjunct in CTO PCI after failure to cross balloon catheters for predilatation. The need for use of RA is low in the current era for CTO intervention.
